# Clinical Study of MEBO Combined with Jinhuang Powder for Diabetic Foot with Infection

**DOI:** 10.1155/2021/5531988

**Published:** 2021-07-13

**Authors:** Hong-Bo Zhan, Qing-Qing Sun, Lei Yan, Jia Cai

**Affiliations:** Department of Burns and Plastic Surgery, Jingjiang People's Hospital of Jiangsu Province, Jingjiang 214500, China

## Abstract

**Background:**

To investigate the efficacy and safety of MEBO combined with Jinhuang powder for the treatment of diabetic foot with infection.

**Methods:**

From August 2015 to August 2019, patients with diabetic foot in our hospital were divided into the treatment group and control group. The treatment group was treated with moist exposed burn ointment (MEBO) combined with Jinhuang powder, while the control group was treated with MEBO only.

**Results:**

After one week of treatment, the effective rate in the treatment group was 100%, and the effective rate in the control group was only 76%. The difference between the two groups was statistically significant (*P* < 0.05). The wound pain score was 2.40 ± 1.38 in the treatment group and 3.76 ± 1.85 in the control group. The difference was statistically significant (*P* < 0.01). After one month of treatment, the effective rate of wound healing was 92.0% in the treatment group and 68% in the control group. The difference between the two groups was statistically significant (*P* < 0.05).

**Conclusion:**

MEBO combined with Jinhuang powder is effective in treating diabetic foot with infection wound.

## 1. Introduction

Diabetes is a group of metabolic diseases characterized by chronic hyperglycemia. Due to insufficient blood supply to the distal extremities, 12%–25% of patients develop diabetic foot ulcers [1–3]. Infection is a high-risk factor to promote the progression and deterioration of diabetic foot. Once it occurs, it will be difficult to control effectively due to the blood supply disorder of the distal limb. The amputation rate of patients with clinical diabetic foot infection was higher than that of patients without infection, and about 80%–85% of diabetic foot patients were forced to choose amputation [4–7].

MEBO is a kind of Chinese patent medicine developed based on the theory of Chinese medicine. Its main drug component is linoleic acid, which is not only an important part of the cell biomembrane but also an indispensable essential fatty acid for cells. It plays an important role in the process of wound tissue repair and healing. MEBO is one of the commonly used drugs for skin ulcers. Topical application can form a protective film on the ulcer surface, prevent the invasion of pathogenic bacteria, maintain the moist environment of the wound, facilitate the discharge of necrotic tissue, and provide a good environment for wound repair. At the same time, it can also improve the blood circulation of the wound and surrounding tissues and promote wound healing [8]. Jinhuang powder is composed of 10 herbs. It is a classic preparation of traditional Chinese medicine surgery. It has the effects of clearing heat and detoxification, promoting blood circulation and reducing swelling. Modern medicine has confirmed that the topical use of the drug can reduce the proportion of leukocytes and neutrophils in peripheral blood, reduce the permeability of blood vessels, and have antiswelling and anti-inflammatory effects. It can improve local blood circulation, effectively control infections, and significantly improve curative effects [9]. Therefore, we speculate that the combined use of MEBO and Jinhuang powder has a better effect on wound healing, but the curative effect of MEBO and Jinhuang powder on wound healing has not been reported.

From August 2015 to July 2018, diabetic foot patients in our department were treated with MEBO combined with Jinhuang powder, and good therapeutic effect was achieved. Therefore, we conducted this study to investigate the efficacy and safety of MEBO combined with Jinhuang powder for the treatment of diabetic foot with infection.

## 2. Materials and Methods

### 2.1. Subjects

From August 2015 to August 2019, patients with diabetic foot in our hospital were divided into the treatment group and control group. The treatment group was treated with MEBO combined with Jinhuang powder, while the control group was treated with MEBO only. This study was conducted in accordance with the Declaration of Helsinki and approved by the ethics committee of our hospital. All participants signed informed consent.

### 2.2. Inclusion and Exclusion Criteria

Inclusive criteria were as follows: (1) patients with Wagner Grade 2, 3, or 4 diabetic foot ulcers; (2) the affected limbs have redness, swelling, heat, pain, and signs of infection; (3) fasting blood glucose control <8 mmol/L; (4) age was older than 18 years; (5) patients who have signed informed consent.

Exclusion criteria were as follows: (1) patients with dysfunction of the heart, liver, kidney, and other organs; (2) ulcers caused by thromboangiitis obliterans and atherosclerosis of lower limbs; (3) patients with poor blood glucose control; (4) patients with systemic infection; (5) patients with hypoproteinemia and moderate and severe anemia; (6) patients whose data were incomplete.

## 3. Methods

The patients were randomly divided into two groups: the combined treatment group and the control group. Both groups were treated with comprehensive medical treatment and surgical treatment. The comprehensive treatment of internal medicine includes the use of insulin to control blood glucose, the selection of appropriate antibiotics to control infection according to the bacterial culture, and drug sensitivity results of wound secretion, as well as vasodilation, anticoagulation, and nutritional nerve therapy. Surgical treatment included removal of necrotic tissue and washing the wound surface and wound cavity with iodophor, hydrogen peroxide, and normal saline. The wound was treated with ultrasonic atomization and continuous low negative pressure suction. In the combined treatment group, Jinhuang powder paste was applied externally to the entire area of the affected foot. After debridement, MEBO gauze was used to fill and cover the wound and cavity, and Vaseline gauze was covered to separate the wound from the Jinhuang powder paste. The dressing was changed twice a day. The control group was not treated with Jinhuang powder paste, and the other treatments were the same as those in the combined treatment group. During treatment, if the patient had the formation of new necrotic softening foci, further treatment was required by incision and drainage procedure. In the later stage, the large granulation wounds were assisted with the point-like (<5 mm) free thin skin graft to promote the wound repair. With reference to the preliminary experiment of this study, the redness and swelling regression rate of patients in the combined treatment group was 100% after one week of treatment, and the redness and swelling regression rate of patients in the control group was about 75% after one week of treatment. The statistical significance test level was *α* = 0.05 (two-sided) and *β* = 0.2, designed according to the sample size 1 : 1, and the sample size was calculated using PASS 11 software. The final sample size was about 24 cases per group.

All procedures were approved by the medical ethics committee of Jinjiang People's Hospital (no. 2015-02-003).

### 3.1. Drugs and Materials

1. MEBO (Shantou MEBO Pharmaceutical Co., Ltd.) (composed of sesame oil and beeswax)
2. Ruyi Jinhuang powder paste (Beijing Tongrentang, Jinhuang powder, proper amount of vinegar made into paste) (composed of trichosanthin, Cortex Phellodendri, rhubarb, turmeric, *Angelica dahurica*, purple *Magnolia officinalis*, tangerine peel, liquorice, *Atractylodes* Rhizoma, and Rhizoma Arisaematis)

### 3.2. Therapeutic Effect Criterion

After one week of treatment, the redness, swelling, and pain of the wound surface were observed as follows: (1) markedly effective: the redness and swelling completely subsided, and the skin plica was obvious; (2) effective: most of the redness and swelling disappeared, and the skin plica was visible; (3) invalid: no obvious change of swelling was observed. The degree of pain was assessed by the numerical rating scale (NRS): (1) mild pain: 1–3 points; (2) moderate pain: 4–6 points; (3) severe pain: 7–10 points.

The wound healing after one month of treatment and the evaluation standard of curative effect refer to the therapeutic effect standard of Guiding Principles for Clinical Research of Chinese New Drugs in the Treatment of Acute Sores formulated and issued by the Ministry of Health [10]. (1) Cure: local swelling disappeared, skin color returned to normal, and the ulcer surface completely healed; (2) markedly effect: local swelling and skin color improved, wound secretion decreased significantly, most of necrotic tissues liquefied and separated, granulation tissue growth was obvious, and the ulcer surface reduced by more than 70%; (3) effective: local swelling and skin color improved, wound secretion decreased, necrotic tissue partially liquefied and separated, the granulation tissue grew, and the ulcer surface reduced by 30%∼70%; (4) no effect: local swelling and skin color did not improve, wound secretion did not significantly reduce, granulation tissue growth was not obvious, and the ulcer surface did not change or expand. The effective rate = (cured + markedly effective + effective)/total number × 100%.

### 3.3. Statistical Methods

We used the software program SPSS 16.0 (IBM, Chicago, USA) to conduct the statistical analysis. The continuous variables of normal distribution were expressed as mean ± standard deviation, the continuous variables of nonnormal distribution were expressed as median (interquartile range (IQR)), and the categorical variables were expressed as frequency (percentage (%)). For two comparisons, each value was compared by *t*-test when each datum conformed to the normal distribution, while the nonnormally distributed continuous data were compared using nonparametric tests. The counting data were tested by chi-square test. A value of *P* < 0.05 was considered statistically significant.

## 4. Results

### 4.1. The General Characteristics

A total of 50 patients with diabetic foot were admitted to our department, including 33 males and 17 females, with an average age of 62.1 years. They were randomly divided into the combined treatment group and the control group with 25 cases in each group. In the combined treatment group, there were 17 males and 8 females, aged 39–86 (65.1 ± 11.7) years; in the control group, there were 16 males and 9 females, aged 37–79 (63.4 ± 11.6) years. There was no significant difference in general data between the two groups, with comparability (*P* > 0.05).

### 4.2. The Clinical Efficacy after One Week

50 patients were treated with this method for one week. The regression rate of wound swelling was 75% in male patients in the control group and 78% in female patients. There was no significant difference between sexes in the group (*P* > 0.05); the swelling and wound regression rate was 100% in both male and female patients in the combined treatment group. There was no significant difference between sexes within the group (*P* > 0.05), so in each group, male and female patients were combined as a whole for comparison between groups.

In the combined treatment group, 18 cases were markedly effective, 7 cases were effective, and no case was ineffective. The effective rate of redness and swelling subsided was 100%. In the control group, 14 cases were markedly effective, 9 cases were effective, 2 cases were ineffective, and the effective rate was 76.0%. The difference between two groups was statistically significant (Table 1). Before treatment, the pain score of male patients in the control group was 4.66 ± 2.29, and the pain score of female patients was 4.72 ± 2.44. There was no significant difference between sexes within the group (*P* > 0.05); after treatment, the pain score of male patients in the control group was 3.72 ± 1.89, and the pain score of female patients was 3.83 ± 1.90. There was no significant difference between sexes within the group (*P* > 0.05). Therefore, the male and female patients in the control group were combined as a whole for the comparison and analysis of the pain scores before and after treatment. The pain score of male patients in the combined treatment group before treatment was 5.09 ± 1.91, and the pain score of female patients was 5.19 ± 2.14. There was no significant difference between sexes within the group (*P* > 0.05); after treatment, the pain score of male patients in the combined treatment group was 2.38 ± 1.28, and the pain score of female patients was 2.44 ± 1.66. There was no significant difference between sexes within the group (*P* > 0.05). Therefore, male and female patients in the combined treatment group were combined as a whole for the comparison and analysis of the pain scores before and after treatment.

Before treatment, the wound pain score of the combined treatment group was 5.12 ± 1.94 points, and that of the control group was 4.68 ± 2.30 points, *P* > 0.05, the difference was not statistically significant, and it was comparable; after one week of treatment, the wound pain score of the combined treatment group was 2.40 ± 1.38 points, and that of the control group was 3.76 ± 1.85 points. The difference was statistically significant (Table 1).

### 4.3. The Clinical Efficacy after One Month

Wounds were treated for one month. The wound healing rate was 69% in male patients in the control group and 67% in female patients. There was no significant difference between sexes in the group (*P* > 0.05); the wound healing rate in the combined treatment group was 94% in male patients and 88% in female patients. There was no significant difference between sexes within the group (*P* > 0.05). Therefore, in each group, male and female patients were combined as a whole for the comparison and analysis of the wound healing rate between the groups.

In the combined treatment group, 5 cases were cured, 6 cases were markedly effective, 12 cases were effective, and 2 cases were ineffective. The effective rate of wound healing was 92.0%. In the control group, 3 cases were cured, 7 cases were markedly effective, 10 cases were effective, and 5 cases were ineffective. The effective rate of wound healing was 68.0%. The difference was statistically significant (*P* < 0.05) (Table 2).

## 5. Discussion

The outcomes of this study presented that the effective rate in the treatment group was significantly higher than that in the control group, and the wound pain score in the treatment group was significantly lower than that in the control group after one week of treatment. After one month of treatment, the effective rate of wound healing in the treatment group was significantly higher than that in the control group.

The World Health Organization (WHO) defines diabetic foot as foot infection, ulcer, and (or) deep tissue destruction related to peripheral vascular diseases and nerve abnormalities in the distal extremity [11], which is also one of the serious complications of disability in diabetic patients. The probability of foot ulcer in diabetic patients is 15 times higher than that in nondiabetic patients [12–14]. About 15% of diabetic patients have foot ulcer [15], which is an important reason for high disability rate of diabetic patients and reducing the quality of life of diabetic patients. At present, the combination of systemic treatment and local treatment is often used. The treatment cycle is long and the medical cost is high, which seriously damages the physical and mental health of patients, affects their quality of life, and increases a great burden on the family and society.

The efficacy of MEBO for diabetic foot has been researched in the industry. It is composed of the matrix and traditional Chinese medicine, and its matrix is composed of sesame oil and beeswax. Sesame oil has small molecular weight, is strongly lipophilic, and has the functions of detoxification, promoting granulation, skin moistening and pain relief, antirancidity, and antisaponification. The main ingredients are Cortex Phellodendri, *Scutellaria baicalensis*, *Lumbricus*, *Coptis*, etc., which have the functions of removing putrefaction and promoting granulation, detoxification and pain relief, promoting blood circulation and removing blood stasis, anti-inflammatory and astringent, and promoting wound healing. MEBO can obviously strengthen the blood circulation of skin capillaries and enhance their resistance; MEBO is a kind of lipophilic paste, which has strong affinity with the skin and wound surface, and can better play the role of clearing heat, detoxification, pain relief, and promoting granulation. Animal experiments show that MEBO can effectively reduce the bacterial metabolic rate, inhibit the growth and reproduction of bacteria, and reduce the virulence and invasiveness of bacteria. MEBO can not only isolate and protect the wound but also drain bacteria, toxins, and metabolites from the inner layer to the outer layer, keep the wound clean, and avoid the continuous infection caused by bacteria. In addition, the important components of MEBO have the function of promoting blood circulation and removing blood stasis, which can improve the microcirculation of the wound, promote the recovery of blood supply, and avoid the accumulation of local oxygen free radicals and the recruitment of inflammatory cells [16–19].

Jinguang powder came from *Orthodox Manual of External Medicine* which was written by Chen Shigong in the Ming Dynasty. It is included in Chinese Pharmacopoeia (Volume I) of 2015 edition. According to the original book, it is composed of trichosanthin, Cortex Phellodendri, rhubarb, turmeric, *Angelica dahurica*, purple *Magnolia officinalis*, tangerine peel, liquorice, *Atractylodes* Rhizoma, and Rhizoma Arisaematis. In the prescription of Jinhuang powder, rhubarb, Cortex Phellodendri, Radix Angelicae Dahuricae, and Radix Glycyrrhizae can promote blood circulation and have the effects of removing dampness and heat, detoxifying and detumescence, promoting granulation, and relieving pain; Rhizoma Arisaematis, *Atractylodes lancea*, Pericarpium Citri Reticulatae, and *Magnolia officinalis* are good for the stomach; the combination of these medicines can clear away heat and toxins, activate blood circulation and remove blood stasis, eliminate swelling, disperse mass, and relieve pain [20, 21]. Modern pharmacological studies show that Jinhuang powder for external use can inhibit bacteria, anti-infection, analgesia, and spasmolysis, relieve local pain, edema, excessive exudate, and secondary infection, and promote the growth of granulation tissue [22].

In our department, MEBO and Jinhuangsan composite paste were used to treat diabetic foot. The method focusing on both internal and external was to clear away necrotic tissue as soon as possible by clearing away heat and toxins, removing putrefaction and promoting granulation, activating blood circulation, and removing blood stasis, so as to activate the ecological tissue between wounds and promote the regeneration of healthy tissues. Meanwhile, due to the specific role of MEBO, some wounds tend to become epithelial cells to heal the wound. It can significantly improve the treatment effect, retain the length of the limb as far as possible, retain the function of the affected limb, reduce the pain of the operation, reduce the adverse reactions, improve the negative psychology of the patients, reduce the cost of treatment, and improve the quality of life of patients to a certain extent. In this study, 2 cases in the combined treatment group failed to heal after one month of treatment. The reason may be due to the repeated severe infection of the wound, the strong virulence of pathogenic bacteria, drug sensitivity of the bacteria culture showing resistance to multiple antibiotics, and poor curative effect caused by difficult control of inflammation.

*Limitations*. There were several limitations in this study. Firstly, this trial was only a single-center trial. Secondly, the sample size was limited. Thirdly, the clinical follow-up was short, and it was necessary to observe the clinical long-term prognosis.

In conclusion, MEBO combined with Jinhuang powder can significantly improve the treatment effect, retain the appearance and function of limbs to the maximum extent, and significantly improve the quality of life of patients. Besides, this treatment had low price, and no obvious toxic and side effects were found recently. Therefore, compared with domestic counterparts, MEBO combined with Jinhuang powder has unique advantages in treating diabetic foot infection.

## Figures and Tables

**Table 1:** The clinical efficacy of MEBO combined with Jinhuang powder after one week on inflammation regression.

Group	Case number	Effectiveness of inflammation regression				Wound pain score (points, ‾*x* ± *s*)	
		Markedly effective	Effective	Ineffective	Effective rate (%)	Before treatment	After treatment
Combined treatment group	25	18	7	0	100.0	5.12 ± 1.94	2.40 ± 1.38
Control group	25	13	6	6	76.0	4.68 ± 2.30	3.76 ± 1.85

*Note*. After one week of treatment, the difference of effectiveness of inflammation regression between two groups was statistically significant (*P* < 0.05). Before treatment, the difference of the wound pain score was not statistically significant (*P* > 0.05); after one week of treatment, the difference of the wound pain score was statistically significant (*P* < 0.01).

**Table 2:** The clinical efficacy of MEBO combined with Jinhuang powder after one month on wound healing.

Group	Case number	Cured	Markedly effective	Effective	Ineffective	Effective rate (%)
Combined treatment group	25	5	6	12	2	92.0
Control group	25	3	5	9	8	68.0

*Note*. After one month of treatment, the effective rate of wound healing was statistically significant (*P* < 0.05).

## Data Availability

The datasets used and analyzed during the current study are available from the corresponding author upon reasonable request.
